# Biomarker roles identification of miR-106 family for predicting the risk and poor survival of colorectal cancer

**DOI:** 10.1186/s12885-020-06863-9

**Published:** 2020-06-03

**Authors:** Qiliang Peng, Yi Shen, Peifeng Zhao, Ming Cheng, Yaqun Zhu, Bo Xu

**Affiliations:** 1 Department of Radiotherapy & Oncology, The Second Affiliated Hospital of Soochow University, Suzhou, China; 2 Institute of Radiotherapy & Oncology, Soochow University, Suzhou, China; 3 Department of Radiation Oncology, The Affiliated Suzhou Science & Technology Town Hospital of Nanjing Medical University, Suzhou, China; 4 Dept. of General Surgery, The Second Affiliated Hospital of Soochow University, San Xiang Road No. 1055, Suzhou, 215004 Jiangsu China

**Keywords:** Colorectal cancer, Biomarker, Meta-analysis, Bioinformatics

## Abstract

**Background:**

Recent studies have extensively investigated the roles of miR-106 in colorectal cancer (CRC). However, the associations and molecular mechanism underlying the roles of miR-106 in CRC remain unclear. We aimed to thoroughly investigate the biomarker roles of miR-106 for predicting the risk and survival outcome in CRC.

**Methods:**

We first conducted a comprehensive meta-analysis to quantitatively evaluate the roles of miR-106 in the diagnosis and prognosis of CRC. Then, we qualitatively explored the biomarker roles of miR-106 in CRC through an integrative bioinformatics analysis.

**Results:**

The results indicated that miR-106 yielded a combined AUC of 0.79 (95% CI: 0.76–0.83), with a pooled sensitivity of 0.50 (95% CI: 0.32–0.68) and a pooled specificity of 0.93 (95% CI: 0.79–0.98) for discriminating CRC cases from normal controls. Moreover, patients with higher expression of miR-106 were significantly associated with shorter disease-free survival (HR: 1.73; 95%CI: 1.23–2.44) and overall survival (HR: 1.39; 95%CI: 1.09–1.77). Finally, gene ontology and pathway analysis demonstrated that miR-106 family was highly involved in the initiation and progression of CRC and indicated the potential molecular mechanism for miR-106 in CRC.

**Conclusions:**

Our results indicated that miR-106 showed promising potential as diagnostic and prognostic biomarker for CRC. Nevertheless, the underlying molecular mechanism of miR-106 family involved in CRC requires further investigation.

## Background

Colorectal cancer (CRC) remains as one of most prevalent malignancies in both developed and developing countries, and it has become a global public health concern due to high mortality [1]. As early symptoms of CRC patients are not typical, most of the CRC cases occur in locally advanced stages when the overall 5-year survival rate are very low. Although a series of predictive methods for diagnosis and prognosis of CRC are available, their clinically application values are limited due to high costs, lack of sensitivity or inconvenience [2]. Thus, new, invasive and more specific methods for early detection and survival prediction are necessary to improve the survival status for CRC patients [3].

MicroRNAs (miRNAs) are a group of small non-coding RNA molecules, which play fundamental roles in regulating gene expression through inhibiting mRNA translation or inducing degradation of the mRNA, and then participate in a wide variety of key physiological processes including cell growth, differentiation, invasion and metastasis [4]. In recent years, numerous studies have suggested that miRNAs may provide a new idea as biomarkers for tumor diagnosis, prognosis and prediction of efficacy [5]. As one of the most common studied miRNA biomarkers, miR-106 has gained great attention as a novel biomarker in cancer detection and survival prediction [6]. Several studies have previously indicated that miR-106 could be specifically used as a promising diagnostic marker for distinguishing CRC patients from normal subjects [7]. Moreover, miR-106 expression level seems to be correlated with CRC patient survival [8]. Nevertheless, different confounding factors, such as sample sources, sample sizes, detection methods, may result in inconsistent and conflicting conclusions. Moreover, the pathological mechanisms of miR-106 involved in CRC are still not fully understood.

Therefore, this study aimed to quantitatively determine the potential biomarker value of miR-106 family and provide a more comprehensive and reliable conclusion on the relationship between miR-106 expression and the diagnosis and prognosis of CRC; in addition, an integrated bioinformatics was performed for uncovering the biomarker functions of miR-106 family at the systems biology level.

## Methods

### Search strategy

Relevant studies published before May 20, 2019 were screened through a search in PubMed, EMBASE, Web of Science, and Cochrane Library databases using the following terms: (“colorectal” or “rectal” or “rectum” or “colon” or “CRC”) and (“cancer”, “carcinoma”, “tumor”, “neoplasm”) and (“microRNA-106” or “miRNA-106” or “miR-106” or “miRNA106”). In addition, we manually examined the references from retrieved articles, including all of the identified studies, reviews, and editorials.

### Inclusion and exclusion criteria for study selection

For inclusion, studies had to meet the following criteria: (1) Investigated the diagnostic or prognostic value of miR-106 family in CRC; (2) Diagnosed of CRC with gold standard; (3) Provided the data to calculate the true positives (TP), false positives (FP), false negatives (FN), true negatives (TN) for diagnosis or HRs and 95% confidence intervals (CIs) for prognosis. For the exclusion, studies were excluded if they: (1) Had nothing to do with our topic; (2) Published as reviews, meta-analysis, letters, or case reports; (3) Provided incomplete data; (4) Were non-English publications.

### Data extraction

Study characteristics and original data were collected independently by two authors from qualified studies, including (1) basic characteristics of the studies including first author, publication year, patient ethnicity, patient age, sample sizes, sample sources, detection method of miR-106 family; (2) diagnostic parameters of miR-106 family, including sensitivity, specificity, and AUC; (3) prognostic parameters of miR-106 family, including follow-up time, the survival outcomes (disease-free survival, DFS; overall survival, OS), HRs and 95% CIs. If HRs and 95% CIs were not reported in the identified articles, they were estimated from Kaplan–Meier curves with methods described by Tierney et al.

### Quality assessment

Two independent investigators evaluated the quality of individual studies respectively based on the QUADAS-2 tool (Quality Assessment of Diagnostic Accuracy Studies 2) for the diagnostic records and the NOS (Newcastle-Ottawa Scale) tool for the prognostic studies [9, 10].

### Statistical methods

For the diagnostic meta-analyses, we evaluated the overall diagnostic results by applying the TP, FP, FN, and TN test results extracted directly from each study for calculating the pooled values including sensitivity, specificity, the positive likelihood ratio (PLR), the negative likelihood ratio (NLR), the diagnostic odds ratio (DOR) with a bivariate random effect-regression model [11]. Meanwhile, we constructed the summary receiver operator characteristic (SROC) curve and calculated the area under the curve (AUC) for quantify the diagnostic performance of miR-106 family [12]. The heterogeneity among studies was examined through the Q test and the I^2^ value. The *P*-value < 0.05 for the Q test or I^2^ ≥ 50% indicated that there was obvious heterogeneity among the selected studies [13]. We applied sensitivity analyses for identifying the possible sources of the heterogeneity. The Deeks’ funnel plot asymmetry test was used to explore the publication bias (*P* value < 0.05 indicated statistically significant).

For the prognostic meta-analyses, HRs and their 95% CIs extracted from studies were pooled for evaluating the prognostic value of miR-106 in CRC. When heterogeneity across studies existed, the random-effect model was conducted for the meta-analysis; otherwise, the fixed-effect model was applied. Subgroup analysis, meta-regression, subgroup and sensitivity analyses were conducted to identify the sources of heterogeneity [14]. In the end, we selected the Begg’s and Egger’s tests to evaluate the potential publication bias among the included studies [15]. All the statistical analyses were performed using STATA 12.0 software.

### Integrated functional enrichment analysis

The biomarker roles of miR-106 may be primarily explained by its transcriptional targets and the involved signal pathways. Therefore, an integrated functional analysis was performed on the targets of miR-106 family. We firstly collected the presumptive targets of miR-106 family from TarBase (v8.0), which is a powerful database of experimentally supported miRNA targets [16]. For targets function annotation, the gene ontology (GO) analysis was analyzed at three different levels: biological processes (BP), cell component (CC) and molecular function (MF) [17]. For pathway enrichment, the predicted targets of miR-106 family were mapped applying the Kyoto Gene and Genome Encyclopedia (KEGG) database [18]. In the present study, the GO and KEGG pathway enrichment analysis were accomplished by online analysis of the Database for Annotation, Visualization and Integrated Discovery (DAVID) tool [19]. Significant enrichment terms were considered as *P*-value < 0.05.

## Results

### Literature search and demographic characteristics

**Fig. 1:**
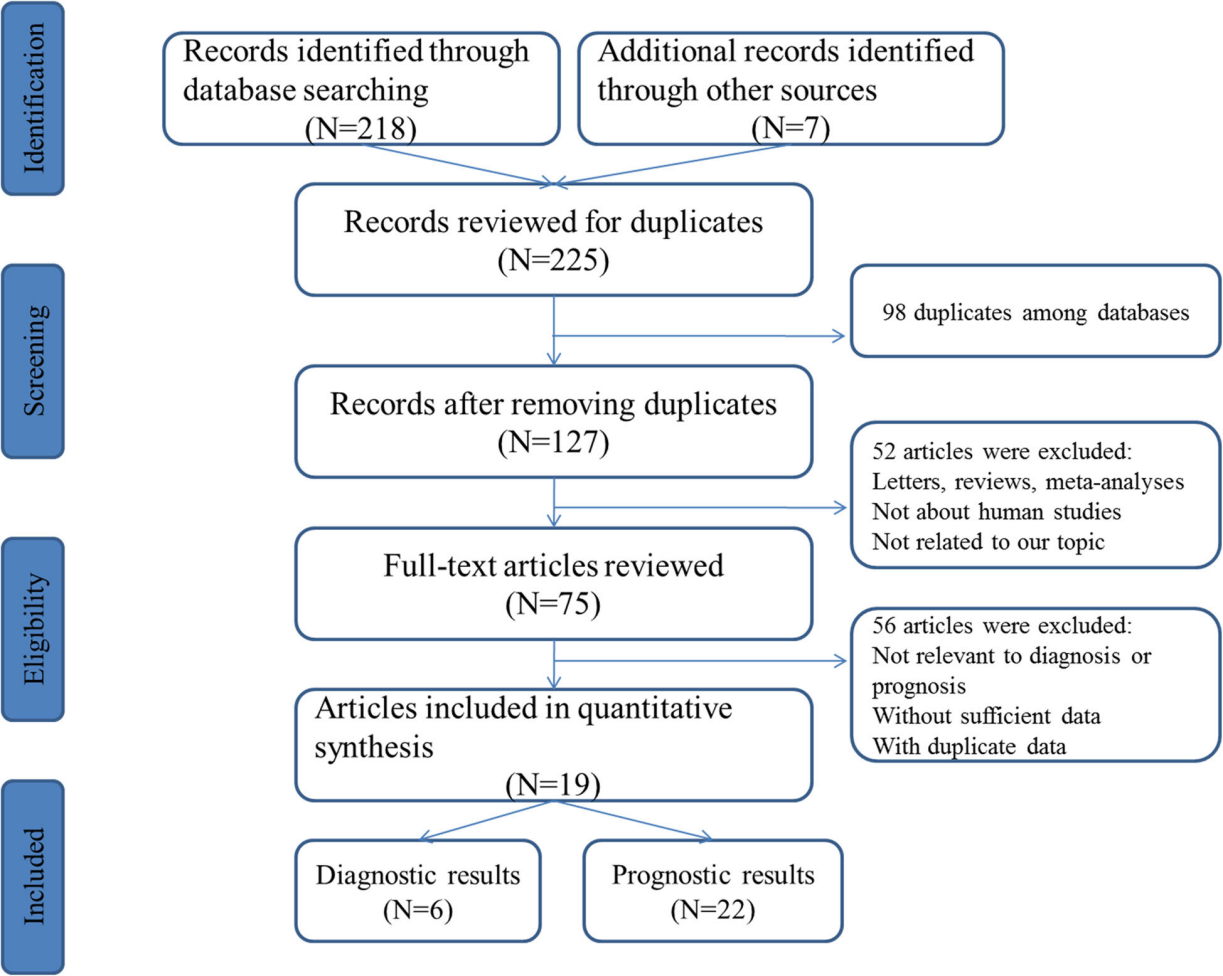
Flow diagram of the study selection process

As shown in Fig. 1, on the basis of initial literature research, a total of 225 qualified articles were involved from the selected databases. According to the inclusion and exclusion criteria, after removing the duplicates and reviewing the texts, 19 articles including 28 studies were utilized for the final analysis, of which 6 studies were about the value of miR-106 family for CRC diagnosis and 22 studies were about CRC prognosis [8, 20–36]. All studies applied quantitative reverse transcription PCR (qRT-PCR) to measure the expression of miR-106 family. The main characteristics of each study were summarized in Table 1 and Table 2. The scores suggested that the majority of enrolled studies had moderately good quality.

**Table 1:** The main features of the included studies for miR-106 family in the diagnosis of CRC

First author	Year	Country	Ethnicity	Case			Control					Sample source	Methods	miRNA	AUC	Sensitivity	Specificity
				M	F	N	Age	M	F	N	Age						
Kuriyama et al. [37]	2012	Japan	Asian	NA	NA	138	NA	NA	NA	126	NA	Feces	RT-PCR	miR-106a	0.826	37.70%	99.20%
Luo et al. [21]	2013	Germany	European	45	35	80	68	60	84	144	62	Plasma	RT-PCR	miR-106b	0.565	19.00%	95.00%
Koga et al. [20]	2013	Japan	Asian	69	48	117	65	66	41	107	60	Feces	RT-PCR	miR-106a	NA	34.20%	97.20%
Chen et al. [22]	2015	China	Asian	60	40	100	60	44	35	79	60	Plasma	RT-PCR	miR-106a	0.605	74.00%	44.40%
Li et al. [23]	2015	China	Asian	113	62	175	57	51	79	130	54	Serum	RT-PCR	miR-106a	0.813	69.00%	83.00%
He et al. [24]	2017	China	Asian	23	19	42	63	24	18	42	60	Plasma	RT-PCR	miR-106a	0.858	74.20%	86.10%

*M* male, *F* female, *N* number, *AUC* area under the curve

**Table 2:** The main features of the included studies for miR-106 family in the prognosis of CRC

First author	Year	Country	Ethnicity	M/F	N	Age	TNM stage	Sample source	miRNA	Methods	Endpoints	Follow-up time (months)	Hazard ratio
Diaz et al. [25]	2008	Spain	European	71/39	110	69	I-IV	Tissue	miR-106a	RT-PCR	DFS	68	0.36(0.17–0.77)
Zhang et al. [28]	2013	China	Asian	79/59	138	65	II	Tissue	miR-106b	RT-PCR	DFS	60	2.36(0.93–6.03)
Zhang et al. [28]	2013	China	Asian	86/51	137	65	I-IV	Tissue	miR-106b	RT-PCR	DFS	60	2.15(0.90–5.11)
Zhang et al. [28]	2013	China	Asian	266/194	460	65	I-IV	Tissue	miR-106b	RT-PCR	DFS	60	2.03(1.34–3.06)
Kjersem et al. [29]	2014	Norway	European	82/68	150	61	I-III	Plasma	miR-106a	RT-PCR	DFS	NA	1.13(0.90–1.41)
Bullock et al. [31]	2015	UK	European	38/12	50	74	I-III	Tissue	miR-106a	RT-PCR	DFS	96	2.91(1.32–6.42)
Li et al. [23]	2015	China	Asian	113/62	175	57	II-III	Serum	miR-106a	RT-PCR	DFS	36	3.02(1.36–6.73)
Zhang et al. [34]	2015	China	Asian	54/39	93	60	I-III	Tissue	miR-106b	RT-PCR	DFS	61	3.47(1.13–10.63)
Yue et al. [33]	2015	China	Asian	42/28	70	65	I-IV	Tissue	miR-106a	RT-PCR	DFS	80	2.21(1.46–4.11)
Caritg et al. [35]	2016	Spain	European	43/26	69	67	II	Tissue	miR-106b	RT-PCR	DFS	140	2.25(0.88–5.75)
Hao et al. [8, 36]	2017	China	Asian	92/46	138	56	I-IV	Tissue	miR-106a	RT-PCR	DFS	60	1.22(0.70–2.12)
Diaz et al. [25]	2008	Spain	European	71/39	110	69	I-IV	Tissue	miR-106a	RT-PCR	OS	68	0.53(0.26–1.07)
Schetter et al. [26]	2008	USA	Caucasians	66/18	84	65	I-IV	Tissue	miR-106a	RT-PCR	OS	68	2.40(1.20–5.10)
Bovell et al. [27]	2013	UK	European	188/193	381	65	I-IV	Tissue	miR-106a	RT-PCR	OS	180	1.42(1.01–2.01)
Kjersem et al. [29]	2014	Norway	European	82/68	150	61	I-III	Plasma	miR-106a	RT-PCR	OS	NA	1.17(0.90–1.52)
Ak et al. [30]	2014	Turkey	European	23/17	40	37	I-IV	Tissue	miR-106a	RT-PCR	OS	24	1.46(0.40–5.37)
Bullock et al. [31]	2015	UK	European	38/12	50	74	I-II	Tissue	miR-106a	RT-PCR	OS	96	2.25(1.00–5.04)
Wang et al. [32]	2015	China	Asian	94/89	183	65	I-IV	Tissue	miR-106b	ISH	OS	80	0.83(0.64–1.07)
Yue et al. [33]	2015	China	Asian	42/28	70	65	I-IV	Tissue	miR-106a	RT-PCR	OS	80	2.07(1.22–3.85)
Zhang et al. [34]	2015	China	Asian	54/39	93	60	I-III	Tissue	miR-106b	RT-PCR	OS	61	3.95(1.05–14.80)
Hao et al. [﻿8﻿]	2016	China	Asian	40/25	65	60	I-IV	Tissue	miR-106a	RT-PCR	OS	60	1.40(1.25–1.93)
Hao et al. [﻿36﻿]	2017	China	Asian	92/46	138	56	I-IV	Tissue	miR-106a	RT-PCR	OS	60	1.87 (1.13–3.09)

*M* male, *F* female, *N* number

### Diagnostic value of miR-106 family in CRC

**Fig. 2:**
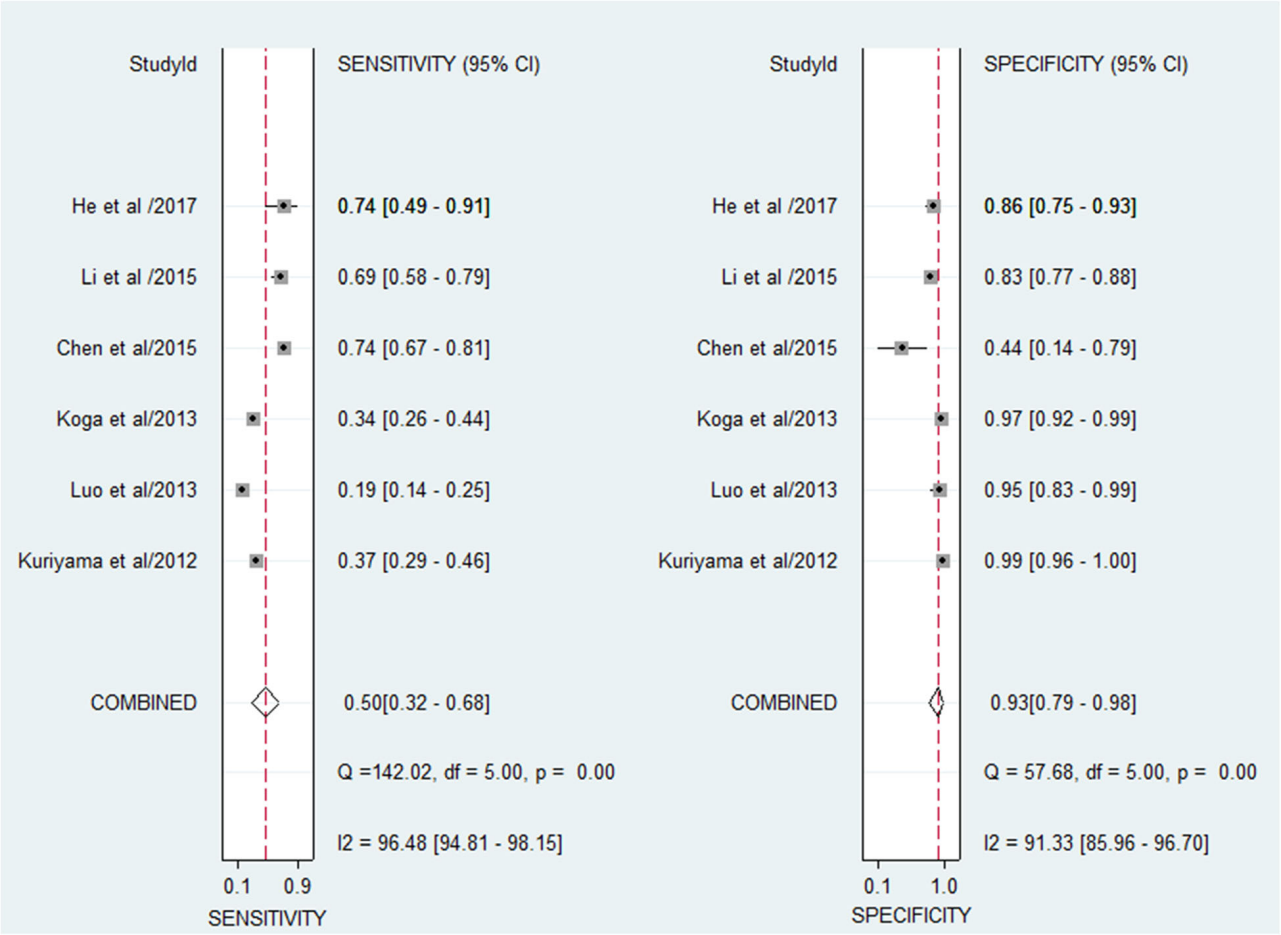
Forest plots of sensitivities and specificities from test accuracy studies in the diagnosis of CRC

**Fig. 3:**
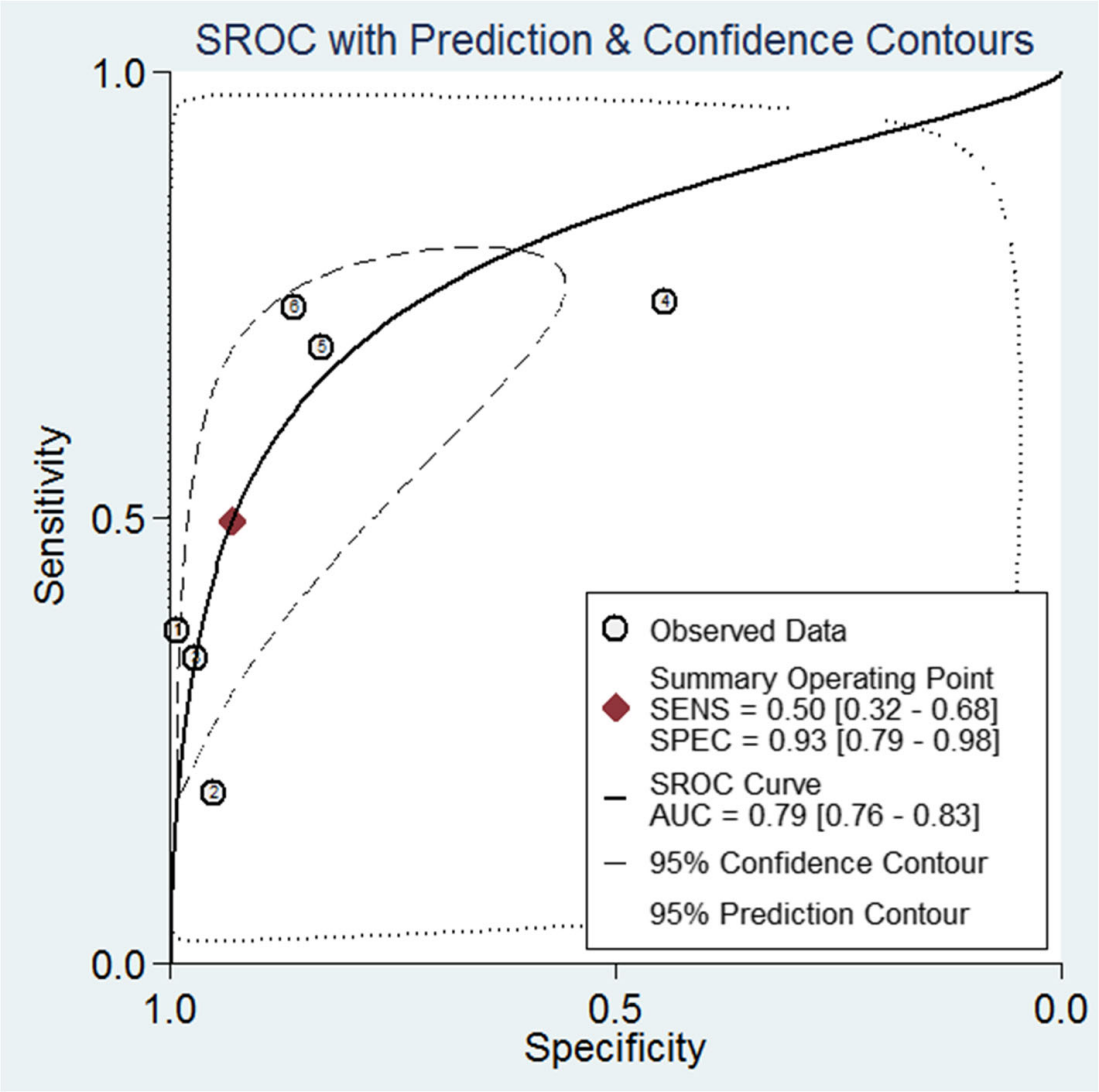
The SROC curves in the diagnosis of CRC

A total of six studies containing 652 patients and 628 normal controls assessed the diagnostic value of miR-106 family for CRC. As shown in Fig. 2, I^2^ values for sensitivity and specificity were 96.48% (95% CI: 94.81–98.15%; *P* < 0.001) and 91.33% (95% CI: 85.86–96.70%; *P* < 0.001), respectively, indicating significant heterogeneity. The pooled sensitivity, specificity, PLR, NLR, and DOR were 0.50 (95% CI: 0.32–0.68), 0.93 (95% CI: 0.79–0.98), 7.1 (95% CI: 3.0–16.9), 0.54 (95% CI: 0.40–0.74), and 13 (95% CI: 6–28), respectively. The SROC curve analyses indicated a relatively high overall diagnostic accuracy, with AUC values of 0.79 (95% CI: 0.76–0.83) for miR-106 family in differentiating CRC from healthy controls (Fig. 3).

**Fig. 4:**
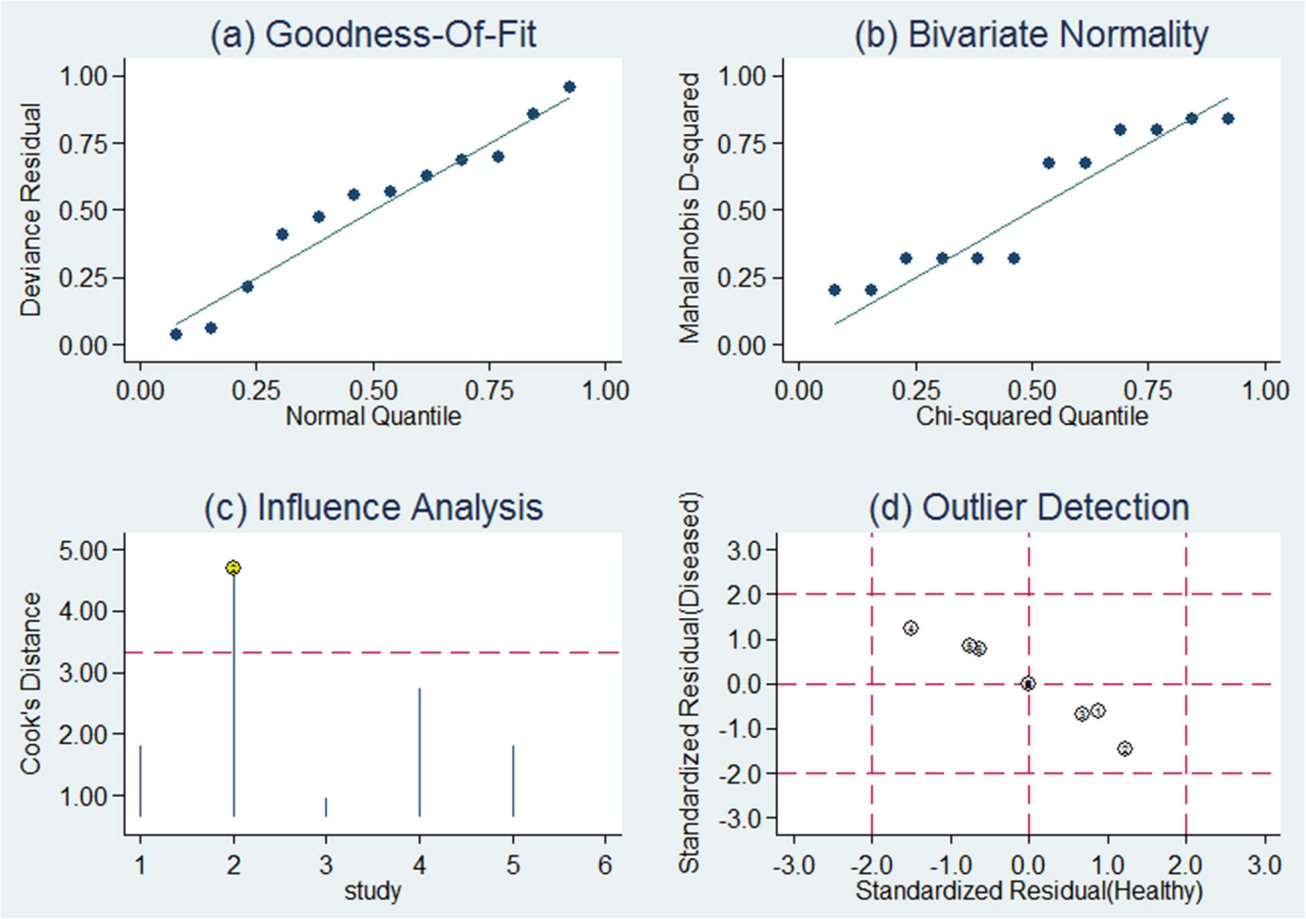
Sensitivity analysis results in the meta-analysis for diagnosis

The goodness of fit and bivariate normality analyses demonstrated that the bivariate meta-analysis model was moderately robust (Fig. 4). Besides that, one outlier study was identified using the method of influence analysis. After omitting it, minimal changes in sensitivity (0.50 vs. 0.56), specificity (0.93 vs. 0.92), PLR (7.1 vs. 6.8), NLR (0.54 vs. 0.48), DOR (13 vs. 14), and AUC (0.79 vs. 0.77) were observed between the overall analysis with and without outlier, suggesting that the study may not have a substantial impact on the combined results. Meta-regression failed to identify the possible source of heterogeneity. Due to the limited number of studies, further analysis about subgroup was not conducted.

Deeks’ funnel plot was applied to assess potential publication bias, and the *P*-value of Deeks’ tests was 0.28, suggesting there was no significant publication bias in this analysis.

### Prognostic value of miR-106 family in CRC

**Fig. 5:**
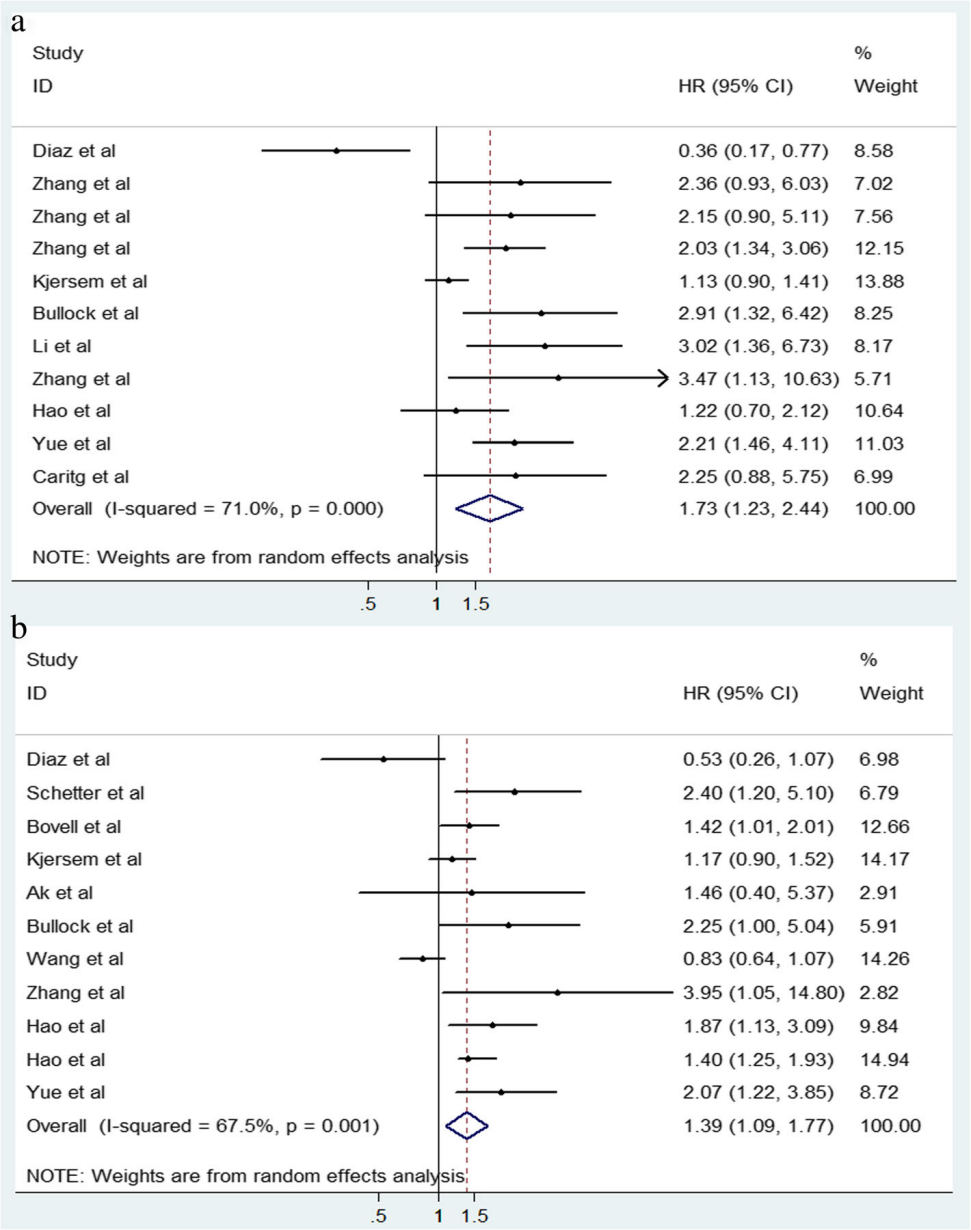
Forest plots of the correlation between miR-106 family expression level and CRC prognosis. **a**. Forest plot of DFS; **b**. Forest plot of OS

A total of 1590 and 1364 patients were enrolled for assessing the prognostic value of miR-106 family in DFS and OS for CRC, respectively. As significant heterogeneity among the enrolled studies was observed (DFS: I^2^ = 71.0%, *P* < 0.001; OS: I^2^ = 67.5%, *P* = 0.001), random-effects models were applied in the analysis for evaluating the prognostic value of miR-106 family in DFS and OS (Fig. 5). According to the pooled analysis, significant associations were identified between miR-106 family and poor DFS (HR = 1.73; 95% CI: 1.23–2.44; *P* = 0.002) and poor OS (HR = 1.39; 95% CI: 1.09–1.77; *P* = 0.008).

We performed subgroup analysis to reveal the potential source of the heterogeneity (Table 3). In the subgroup of DFS outcomes, we found that the predictive role of miR-106 family was more significant for miR-106b (HR = 2.19; 95% CI: 1.61–3.00) than miR-106a (HR = 1.44; 95% CI: 0.88–2.35). In addition, the association was more significant in Asian patients (HR = 2.02; 95% CI: 1.60–2.57) than in European patients (HR = 1.24; 95% CI: 0.59–2.60). In the subgroup of OS outcomes, high miR-106a levels were significantly associated with a worse OS in CRC (HR = 1.45; 95% CI: 1.16–1.80). And for miR-106b, however, this association was not statistically significant (HR = 1.57; 95% CI: 0.35–7.08). When grouped according to the ethnicity, combined HR of Asian patients and non-Asian patients were 1.50 (95% CI: 1.00–2.24), and 1.33 (95% CI: 0.94–1.88), respectively, indicating that miR-106 family were more predictive as indicators of poor prognosis in Asian CRC patients.

**Table 3:** Results of subgroup and meta-regression analyses in the prognostic meta-analysis

Outcome	Subgroup	Studies	HR (95%CI)	***P***-value	Heterogeneity (I^**2**^)	P_**heterogeneity**_	Meta-regression (***P***-value)
DFS	**miRNA classification**						*P* = 0.237
	miR-106a	6	1.44(0.88–2.35)	*P* = 0.151	79.9%	P < 0.001	
	miR-106b	5	2.19(1.61–3.00)	P < 0.001	0	*P* = 0.938	
	**Ethnicity**						*P* = 0.322
	Asian	7	2.02(1.60–2.57)	*P* = 0.569	81.7%	P = 0.001	
	Non-Asian	4	1.24(0.59–2.60)	P < 0.001	0	*P* = 0.507	
OS	**miRNA classification**						*P* = 0.584
	miR-106a	9	1.45(1.16–1.80)	P = 0.001	48.6%	*P* = 0.049	
	miR-106b	2	1.57 (0.35–7.08)	*P* = 0.555	80.6%	*P* = 0.023	
	**Ethnicity**						*P* = 0.648
	Asian	5	1.50(1.00–2.24)	*P* = 0.050	79.1%	P = 0.001	
	Non-Asian	6	1.33(0.94–1.88)	*P* = 0.107	56.8%	*P* = 0.041	

We also performed meta-regression analysis to investigate the sources of heterogeneity. The meta-regression results revealed that the heterogeneity between studies evaluating miR-106 family in DFS and OS may not be induced by ethnicity (*P* > 0.05), miRNA classification (*P* > 0.05), and sample size (*P* > 0.05).

**Fig. 6:**
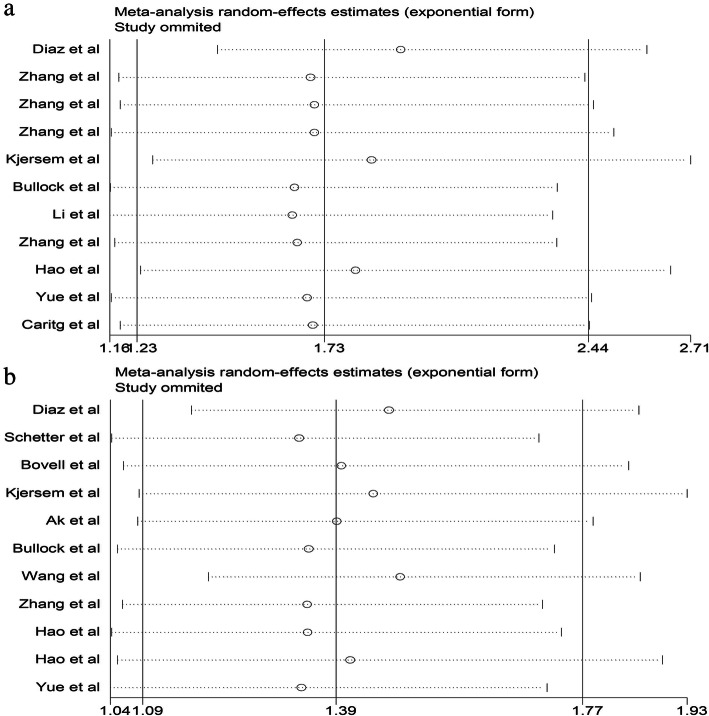
Sensitivity analyses in the meta-analysis for prognosis. **a**. Sensitivity analysis for DFS; **b**. Sensitivity analysis for OS

Sensitivity analysis was further carried out by sequentially omitting individual studies, indicating that there was no obvious influence of individual study on the pooled HRs, no matter which article was removed (Fig. 6).

**Fig. 7:**
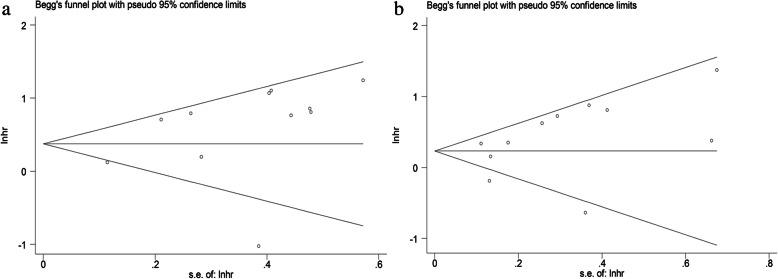
Begg’s funnel plots for evaluating publication bias in the meta-analysis for prognosis. **a**. Funnel plot of the studies for DFS. **b**. Funnel plot of the studies for OS

Finally, potential publication bias was evaluated with Begg’s funnel plot and Egger’s test (Fig. 7). The *P*-value of 0.15 and 0.21 indicated no significant publication bias exist in the analysis for assessing the association of DFS, OS and miR-106 family expression.

### Function exploration of miR-106 family in CRC

We further performed an integrated bioinformatics analysis to explore the function of miR-106 family and to answer the question why miR-106 family could possess the biomarker characteristics in the diagnosis and prognosis of CRC. Of great importance, we believe that if miR-106 family could play an important role in the occurrence and development of CRC, the genes regulated by miR-106a and miR-106b should also play a part in CRC. We first retrieved the target genes of miR-106a and miR-106b from the powerful TarBase database. Then the gene lists were uploaded to DAVID tool to gain functional enrichment information.

In the present study, we mainly concentrated on the top 10 significantly enriched terms for further discussion. The GO enrichment analysis results of miR-106a and miR-106b were presented at Table 4 and Table 5, respectively. The enrichment results given by GO analysis of miR-106a indicated that the GO terms for BP level associated with the target genes were including phosphorylation, cell cycle arrest, regulation of mitotic cell cycle and transforming growth factor beta (TGF-β) receptor signaling pathway. The associated CC level for miR-106a included cytoplasm, nucleus, nucleoplasm and nucleolus. The significant GO terms for MF level were closely relevant to binding function and enzyme activity. And for miR-106b, at the BP level, the most significant terms were highly linked with transcription and ubiquitination. At the CC level, the enriched terms were closely relevant to nucleoplasm, nucleus and nucleolus. At the MF level, most enriched terms were also mainly concentrated on binding function and enzyme activity.

**Table 4:** GO enrichment analysis results for miR-106a

BP		
GO terms	Genes	***P***-value
Peptidyl-serine phosphorylation	24	7.10E-07
Cell cycle arrest	16	2.07E-06
Regulation of mitotic cell cycle	10	1.51E-05
Negative regulation of TGF-βreceptor signaling pathway	12	1.26E-04
Cellular response to amino acid stimulus	11	1.50E-04
Protein ubiquitination involved in ubiquitin-dependent protein catabolic process	20	1.61E-04
Protein autophosphorylation	19	2.91E-04
Transforming growth factor beta receptor signaling pathway	12	7.78E-04
Plus-end-directed vesicle transport along microtubule	4	1.37E-03
Mitochondrial genome maintenance	5	1.43E-03
**CC**		
**GO terms**	**Genes**	***P*-value**
Cytoplasm	269	1.64E-14
Nucleus	255	2.89E-10
Nucleoplasm	136	1.40E-07
Membrane	96	6.43E-07
Focal adhesion	41	3.48E-05
Nucleolus	65	5.42E-05
CCR4-NOT complex	7	6.55E-05
Centrosome	39	2.15E-04
Transcription factor complex	25	2.26E-04
Cytoskeleton	17	2.79E-04
**MF**		
**GO terms**	**Genes**	***P*-value**
ATP binding	124	1.47E-06
Protein serine/threonine kinase activity	33	4.69E-05
Poly(A) RNA binding	90	2.25E-04
Ubiquitin-protein transferase activity	23	4.79E-04
RNA binding	33	6.95E-04
Thiol-dependent ubiquitin-specific protease activity	14	7.26E-04
Znc ion binding	93	1.11E-03
1-phosphatidylinositol binding	6	1.30E-03
Receptor signaling protein serine/threonine kinase activity	11	2.78E-03
DNA binding	59	4.38E-03

*GO* gene ontology, *BP* biological process, *CC* cellular component, *MF* molecular function

**Table 5:** GO enrichment analysis results for miR-106b

BP		
GO terms	Genes	***P***-value
Transcription, DNA-templated	309	4.29E-12
Protein ubiquitination	82	1.53E-10
Negative regulation of transcription from RNA polymerase II promoter	135	3.84E-10
Viral process	66	4.52E-08
Protein polyubiquitination	47	5.79E-08
Protein ubiquitination involved in ubiquitin-dependent protein catabolic process	41	1.15E-07
Cellular response to DNA damage stimulus	49	4.09E-07
Cell-cell adhesion	59	4.12E-07
Positive regulation of transcription, DNA-templated	94	7.99E-07
Positive regulation of transcription from RNA polymerase II promoter	157	8.43E-07
**CC**		
**GO terms**	**Genes**	***P*-value**
Nucleoplasm	509	4.53E-41
Nucleus	817	4.14E-36
Cytoplasm	762	8.37E-28
Cytosol	512	2.68E-22
Membrane	337	2.06E-13
Nucleolus	153	6.39E-11
Cell-cell adherens junction	70	8.52E-09
Intracellular membrane-bounded organelle	100	1.50E-07
Midbody	35	3.50E-07
Perinuclear region of cytoplasm	103	3.83E-06
**MF**		
**GO terms**	**Genes**	***P*-value**
Protein binding	1251	6.93E-44
Poly(A) RNA binding	211	5.55E-15
Ubiquitin-protein transferase activity	81	5.55E-12
Ubiquitin protein ligase binding	70	2.73E-10
Ubiquitin protein ligase activity	52	5.74E-10
Cadherin binding involved in cell-cell adhesion	67	7.22E-09
Transcription factor activity, sequence-specific DNA binding	155	1.02E-06
DNA binding	246	1.35E-06
ATP binding	221	3.40E-06
Protein serine/threonine kinase activity	72	4.18E-06

*GO* gene ontology, *BP* biological process, *CC* cellular component, *MF* molecular function

The top 10 KEGG pathway enrichment analysis results of miR-106a and miR-106b were listed in Table 6. The enrichment analysis suggested that the targeted genes of miR-106a were significantly involved in FoxO signaling pathway, focal adhesion, colorectal cancer, pathways in cancer and MAPK signaling pathway. The results of enriched KEGG pathway analysis revealed that the targets of miR-106b were significantly clustered in cell cycle, FoxO signaling pathway, pathways in cancer, RNA degradation and some other diseases including prostate cancer and chronic myeloid leukemia.

**Table 6:** KEGG pathway analysis for miR-106 family

A, miR-106a		
Pathway	Genes	***P***-value
FoxO signaling pathway	28	3.62E-08
Focal adhesion	35	3.54E-07
Hepatitis C	26	8.70E-07
Colorectal cancer	15	5.81E-05
Endocytosis	32	1.44E-04
Prostate cancer	17	1.44E-04
Pathways in cancer	45	1.64E-04
Adherens junction	15	1.83E-04
Pancreatic cancer	14	2.75E-04
MAPK signaling pathway	32	4.58E-04
**B, miR-106b**		
**Pathway**	**Genes**	***P*-value**
Cell cycle	33	2.14E-06
Protein processing in endoplasmic reticulum	39	8.97E-06
FoxO signaling pathway	33	1.25E-05
Pathways in cancer	70	3.17E-05
Prostate cancer	24	4.63E-05
Hepatitis C	31	7.36E-05
RNA degradation	21	1.57E-04
Hepatitis B	32	1.61E-04
Chronic myeloid leukemia	20	1.82E-04
Endocytosis	46	1.87E-04

## Discussion

Early diagnosis and dynamic monitoring after treatment of CRC is a well-established consensus for patients to receive proper therapeutic treatment and can improve patient survival. Accumulating studies have found miR-106 family as a promising biomarker with key roles in the pathogenesis and tumorigenesis of CRC. Nevertheless, different studies reported with inconsistent results. Thus, by using meta-analysis and bioinformatics analysis, we aimed to obtain a comprehensive understanding of associations between miR-106 expression and diagnosis and prognosis of CRC patients.

It was revealed from the diagnostic meta-analysis that miR-106 family was 50% sensitive and 93% specific in distinguishing between CRC and normal controls (AUC: 0.79). The PLR, NLR, and DOR were 7.1, 0.54, and 13, respectively. The overall results suggested that miR-106 family may serve as a promising diagnostic biomarker in the CRC detection with a moderate accuracy. However, there is a long way to go before the application of miR-106 family into clinical as they still have insufficient power to accurately detect and diagnose CRC.

Prognostic meta-analysis indicated that patients with high levels of miR-106 family were related to poorer survival than those with low expression levels. The predictive roles were more significant for miR-106a in OS and miR-106b in DFS. Meanwhile, the results indicated that miR-106 family was more predictive as biomarker of poor prognosis in Asian CRC patients. In all, pooled HR values of DFS and OS correlated with miR-106 expression for CRC patients, which revealed that miR-106 could be an independent risk factor for prognosis and may be used to monitor the therapeutic effects of radical resection or systemic adjuvant therapy.

As miRNAs contribute to tumorigenesis by regulating gene expression in various biological activities, we performed a functional enrichment analysis of the target genes of miR-106 family to explore their potential mechanisms involved in the initiation and progression of CRC. Published literatures revealed that the biological processes associated with miR-106 family including phosphorylation, cell cycle and TGF-β receptor signaling for miR-106a and transcription, ubiquitination for miR-106b, were highly related to the initiation and progression in CRC as they were significantly involved in regulating numerous cellular activities, such as apoptosis, proliferation, differentiation, gene regulation, metabolism, and metastasis [38–41]. Enrichment GO analysis also suggested that miR-106a and miR-106b were both mostly correlated with the vital cell components including nucleus, nucleoplasm and nucleolus, which have been demonstrated to be associated with the proliferation and invasion of CRC [42]. For MF, the targets of miR-106a and miR-106b were mainly linked with the binding function and enzyme activity, which has also been proved to be involved in the development and classification of CRC [43, 44].

What’s more, the KEGG pathway enrichment analysis revealed that some important pathways associated with miR-106a and miR-106b might take part in the pathogenesis of CRC according to literature exploration. For example, the colorectal cancer pathway directly proved that miR-106 indeed participated in the occurrence and development of CRC. FoxO signaling pathway, which is the central regulator of cellular homeostasis and tumor suppressors in human cancers, plays a central role in diverse physiological processes from development, cell signaling, and tumorigenesis to cell metabolism [45]. With regard to the MAPK signaling pathway, its imbalance in expression is associated with various cellular activities involved in cancer progression, including proliferation, apoptosis and immune escape [46]. Cell cycle, perhaps the most important pathway with a high correlation with colorectal carcinogenesis, plays its role through regulating cell growth, differentiation, apoptosis, cancer development and metastasis [47]. There is mounting evidence to indicate that activation of this pathway contributes to the pathogenesis, progression, and oncogenic behavior of human CRC [48]. About the focal adhesion pathway, accumulating new evidence supports the concept that it plays important roles in the invasion and metastasis of a variety of tumors and is correlated with the medicine resistance of certain tumors [49]. RNA degradation is a highly crucial process in the regulation of gene expression. The abnormal activation of this pathway may contribute to the physiological alterations towards carcinogenesis [50]. The functional enrichment results indicated the preliminary roles of miR-106 family in the occurrence and development of CRC, which should be evaluated and validated by further mechanistic studies.

There is still a long way for the application miR-106 into clinical practice. Although qRT-PCR was the most common method for detecting miR-106 expression, in situ hybridization (ISH) was also used in some studies. Both qRT-PCR and ISH may provide a reliable evidence for cancer detection and have their own disadvantages as well. However, heterogeneity may result from different laboratories using different methods to quantify miR-106. There is thus a great need for further studies to reach agreement on the procedure used for normalization. Various sample sources (tissue, plasma, serum, feces) have presented the potential for detecting miR-106. We supposed that tissue miR-106 could be applied for predicting the survival outcome and circulating miR-106 may act as an auxiliary marker, monitoring the level of miR-106 in the body. For clinical purpose, it requires more studies and analyses to investigate the diagnostic value of miR-106 in specific specimen for detecting CRC.

Several limitations of our study should be considered before interpreting the results. Firstly, the lack of access to the original data from the studies may hinder the integrated investigation of the associations between miR-106 expression and the diagnosis and prognosis of CRC, which is the main problem. Secondly, there were only six studies included in the evaluation of diagnosis value of miR-106 family, so the statistical power of our study may be constrained. Thirdly, no study was conducted in Africa, which may also restrict the research extent.

Despite these limitations, our study had several important strengths. To begin with, a relatively thorough systematic search was carried out, and the biomarker roles of miR-106 family in the diagnosis and prognosis of CRC were independently assessed and validated. Then, we not only quantitatively demonstrated that miR-106 family may have potential to serve as a promising and non-invasive biomarker for CRC, but qualitatively indicated the underlying roles of miR-106 family in the occurrence and development of CRC. Besides, our study also provided some interesting information, which deserved further investigation.

## Conclusions

In summary, our integrated analysis identified miR-106 family to be highly involved in the initiation and progression of CRC and could be potential and promising biomarker for the preliminary screening and survival prediction of CRC. The results would be helpful for promoting miR-106 family into the clinical application as biomarkers for the diagnosis and prognosis of CRC. However, more large-scale prospective studies are required to clarify the diagnostic efficiency and prognostic value of miR-106 in CRC. Moreover, the specific mechanisms of miR-106 family in tumorigenesis of CRC need to be further explored by biological experiments.

## Data Availability

Data is available from the corresponding author upon reasonable request.

## Abbreviations

CRC: Colorectal cancer
AUC: Area under the curve
TP: True positive
FP: False positive
FN: False negative
TN: True negative
PLR: Positive likelihood ratios
NLR: Negative likelihood ratios
DOR: Diagnostic odds ratio
CI: Confidence interval
SROC: Summary receiver operator characteristic
QUADAS-2: Quality Assessment of Diagnostic Accuracy Studies 2
GO: Gene ontology
KEGG: Kyoto Encyclopedia of Genes and Genomes
DAVID: Database for Annotation, Visualization, and Integrated Discovery
BP: Biological process
CC: Cellular component
MF: Molecular function
